# Supplementary material for: Insights into the zebrafish left–right organizer's centrosomes and cilia via volume electron microscopy
Source: Biol Open. 2026 Mar 23;15(3):bio062489. doi: 10.1242/bio.062489 (PMC13054934; doi:10.1242/bio.062489)
Supplement: Supplementary information [file biolopen-15-062489-s1.pdf]

Table S1. Statistical analysis.

Figure	Category	n embryo	n Clutch	Statistical Test	Parameters	Result	p-value
1E	Ac-tub: Gamma-tub	Clutch 1=13<br>Clutch 2=10<br>Clutch 3=6	n=3	N/A	N/A	N/A	N/A
	Ac-tub: IFT88	Clutch 1=8<br>Clutch 2=5<br>Clutch 3=5					
	Gamma-tub: Rootletin	Clutch 1=8<br>Clutch 2=10<br>Clutch 3=5					

Array tomography

Figure	Category	n structure	n embryo
3E	Mother centriole plus daughter centriole	n=67 cilia	n=1
	Mother centriole		
	No centriole		
4C	Rootlet	n=59 centrosome	n=1
	DA		
	SDA		
	DA and SDA		

Biology Open • Supplementary information

	CPMs		
	CaDR		
5H	CaDVs	n=67 cilia	n=1
	CaVs		
	Ciliary pocket		
	NIVB		

Biology Open • Supplementary information

Table S2. SUPPLEMENTARY KEY RESOURCE TABLE

Reagent or resource	Source	Identifier
**Antibodies**		
Anti-IFT88	Proteintech	13967-1-AP; RRID: AB_2121979
Rootletin	Fisher scientific	ABN1686MI
Acetylated Tubulin	Sigma Aldrich	T6793: RRID: AB_477585
γ-tubulin	Sigma Aldrich	T5192; RRID: AB_261690
Anti-GFP (Chicken)	GeneTex	GTX13970: AB_371416
Anti-GFP (Rabbit)	Molecular Probes	A-11122: AB_221569
Alexa Fluor Anti-Chicken 488	Fisher scientific	A11039
Alexa Fluor Anti-Rabbit 488	Life Technologies	A21206; RRID: AB_2535792
Alexa Fluor Anti-Rabbit 568	Life Technologies	A10042; RRID: AB_2534017
Alexa Fluor Anti-Rabbit 647	Life Technologies	A31573; RRID: AB_2536183
Alexa Fluor Anti-Mouse 488	Life Technologies	A21202; RRID: AB_141607
Alexa Fluor Anti-Mouse 568	Life Technologies	A10037; RRID: AB_2534013
Alexa Fluor Anti-Mouse 647	Life Technologies	A31571; RRID: AB_162542
**Chemicals, Peptides, and Recombinant Proteins**		
DAPI	Sigma Aldrich	D9542-10mg
Agarose	Thermo Fischer	16520100
BSA	Fisher Scientific	BP1600-100
BIO BASIC Maxi Prep Kit	BIO BASIC	9K-0060023
Dimethylsulphoxide	Fisher Scientific	BP231-100
Paraformaldehyde	Fisher Scientific	O4042-500
Phosphate Buffered Saline	Fisher Scientific	10010023
Molecular Probes Prolong Gold Antifade mount	Fisher Scientific	P36934

Biology Open • Supplementary information

Triton X-100	Fisher Scientific	BP151500
Tween 20	ThermoFischer	BP337500
Sodium Chloride	Fisher Scientific	BP358
NEBuilder HiFi DNA assembly Cloning Kit	New England BioLabs	E5520S
mMESSAGE mMACHINETMSP6	Invitrogen	AM1340
OneTaq One-Step RT-PCR Kit	New England Biolabs	E5315S
**Reagent or resource**	**Source**	**Identifier**
Karnovsky's fixative<br>20% Formaldehyde<br>10% Glutaraldehyde	EMS<br>EMS	15713<br>16120
Low-melt agarose	Millipore Sigma	A9045-10G
4% Osmium tetroxide aqueous solution	EMS	19170
Sodium cacodylate trihydrate	EMS	12310
Potassium ferricyanide	EMS	20150
Uranyl acetate	EMS	22400
Lead aspartate solution<br>Lead nitrate<br>Aspartic acid	EMS<br>Millipore Sigma	17900<br>A8949-25G
Ethanol	EMS	15055
Propylene oxide	Millipore Sigma	110205-500ML
Polybed 812 resin	Polysciences<br>Polysciences<br>Polysciences<br>EMS	Poly/bed® 812 embedding media: 08791<br>Nadic Methyl Anhydride (NMA): 00886<br>Dodecenylsuccinic anhydride (DDSA): 00563<br>Benzyldimethylamine (BDMA): 11400
**Equipment and consumables**		
35 mm Dish| No.1.5. coverslip| 20 mm Glass Diameter	MatTek Corporation	P35G-1.5-20-C
40 mm x 22 mm No. 1 8-12 Ohm/sq ITO coverslip	SPI Supplies	06497-AB
Silicon wafer	EMS	71893-07
Conductive copper tape	EMS	77802

Biology Open • Supplementary information

45 Ultra Diamond knife	Diatome	25-US
ARTOS Ultramicrotome	Leica	https://www.leica-microsystems.com/products/sample-preparation-for-electron-microscopy/p/artos-3d/
**Experimental models, organisms, and strains**		
Zebrafish	Dasgupta and Amack, 2016 [1]	Tg (Sox17:GFP-CAAX)sny101
**Software and algorithms**		
3D Dragonfly software	ORS Dragonfly	https://dragonfly.comet.tech/
ATLAS 5 Array Tomography software (Fibics/Zeiss)	Fibics Inc	http://www.fibics.com/
Python-based scripts	CCR volume EM	https://crtp.ccr.cancer.gov/vem/
ImageJ/FIJI	NIH and Laboratory for Optical and Computational Instrumentation	https://imagej.net/Fiji
IMARIS, Bitplane	Oxford Instruments	https://imaris.oxinst.com/
PRISM9	GraphPad	https://www.graphpad.com/scientific-software/prism/
LAS-X Software	Leica Microsystems	https://www.leica-microsystems.com/products/microscope-software/p/leica-las-x-ls/
VisiView	Visitron	https://www.visitron.de/products/visiviewr-software.html

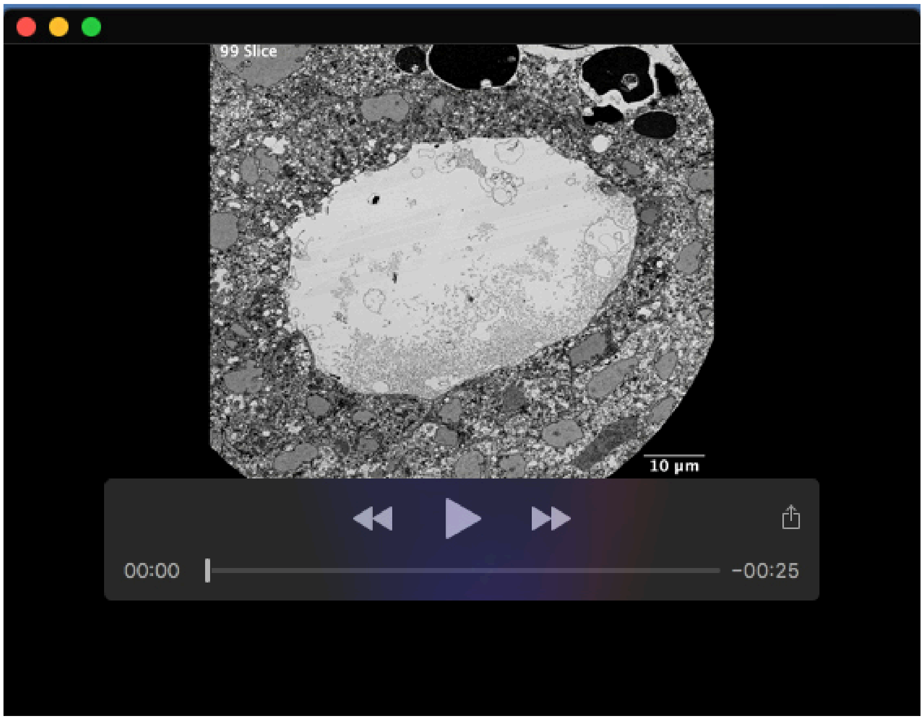

Movie 1. Workflow overview for vEM imaging of the Kupffer's Vesicle (KV). Video shows a series of 406 vEM slices through the zebrafish KV.

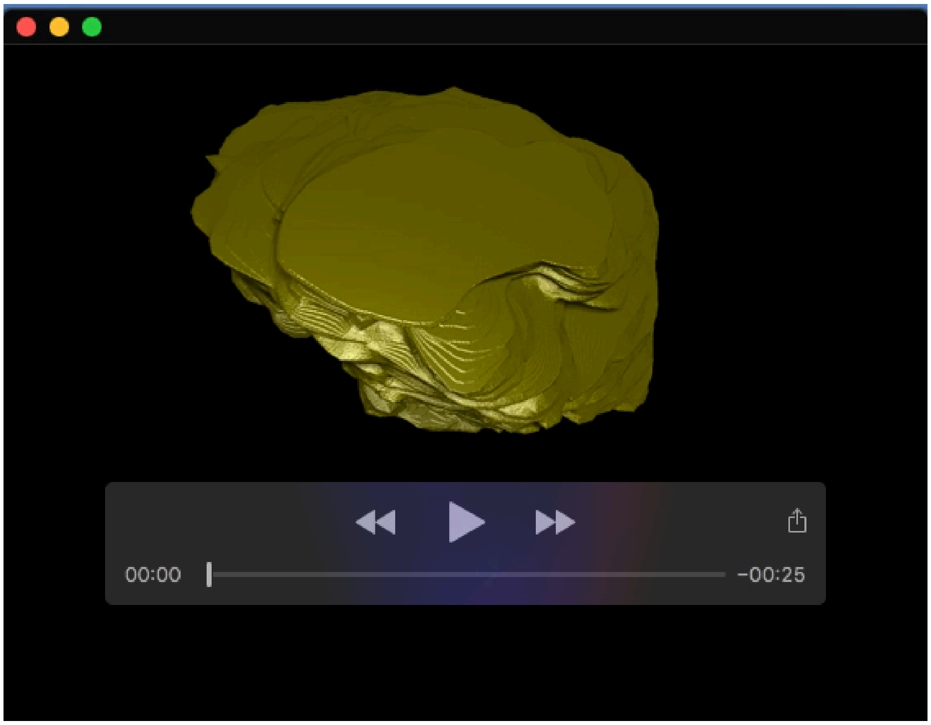

Movie 2. vEM of the zebrafish KV reveals that most cilia associate with both mother and daughter centrioles, but a subset lack one or both. Video shows a 3D segmentation of entire KV. KV region (green), lumen (gray), nuclei (multi-colored), cilia (cyan), centrosomes (orange)
